# Stable Encapsulation and Responsive Release of Dyes via Noncovalent Molecular Lock Strategy: A Case Study of Rhodamine B Based Fluorescent Hydrogel Microspheres

**DOI:** 10.3390/polym18040493

**Published:** 2026-02-16

**Authors:** Shuo Meng, Chuanyu Dang, Xiaoyong Qiu, Jianhua Chen, Ruiheng Yao, Yuquan Wang, Luxing Wei, Jun Huang, Xiaolai Zhang

**Affiliations:** 1 Key Laboratory of Colloid and Interface Chemistry of the Ministry of Education, School of Chemistry and Chemical Engineering, Shandong University, Jinan 250100, China; upmovm8718@163.com (S.M.); xyqiu@sdu.edu.cn (X.Q.); 2 Shandong Kunda Biotech Co., Ltd., Linyi 276400, China; dcyisme@163.com; 3 Beijing Fleming Technology Co., Ltd., Beijing 102600, China; chenjianhua@bjfleming.com; 4 Center for Advanced Jet Engineering Technologies (CaJET), Key Laboratory of High Efficiency and Clean Mechanical Manufacture of Ministry of Education, School of Mechanical Engineering, Shandong University, Jinan 250061, China; 202414389@mail.sdu.edu.cn (R.Y.); 202414391@mail.sdu.edu.cn (Y.W.); wlx12033714@163.com (L.W.); jun.huang@email.sdu.edu.cn (J.H.)

**Keywords:** microfluidic, noncovalent interactions, stable encapsulation, responsive release, tracing

## Abstract

Hydrogel fluorescent microspheres function as versatile tracers with applications spanning across biomedicine, complex plasma systems, hydrodynamics, and drug delivery. However, the controlled release of fluorescent material in hydrogel microspheres is challenging to achieve. The fluorescent hydrogel microsphere (namely poly(ethylene glycol) diacrylate@rhodamine B-tannic acid, PEGDA@RhB-TA) was fabricated by incorporating tannic acid and RhB into PEGDA microspheres. The stable encapsulation and responsive release of RhB can be achieved by leveraging the non-covalent interactions between TA and RhB. RhB was stably encapsulated within PEGDA microspheres through noncovalent interactions (hydrophobic interactions, hydrogen bonding, π–π, and ion–π interactions) between RhB and TA. Both molecular dynamics simulations by GROMACS and experimental results confirmed the noncovalent binding mechanisms between RhB and TA. The microspheres retained RhB following 24 h immersion in a highly concentrated salt solution (1 M NaCl) and exhibited minimal RhB release (7.1%) under heating at 80 °C for 24 h. However, PEGDA@RhB-TA microspheres underwent rapid RhB release in a 50% *v*/*v* ethanol–water solution, liberating 73% of the encapsulated dye within 24 h. TA within the PEGDA@RhB-TA microsphere acts as a molecular lock by forming non-covalent interactions with RhB, significantly enhancing the stability of encapsulated RhB, and enabling the responsive release of RhB under specific conditions. Upon introduction into a microfluidic chip, PEGDA@RhB-TA microspheres enable the calculation of flow velocity through position tracking using high-speed camera imaging and fluorescence microscopy. These microspheres overcome the dual challenges of tracer stability and controlled release, making them suitable for fluid tracing and measuring flow rates.

## 1. Introduction

Hydrogel microspheres integrate the high-water content characteristic of hydrogels with the microscale features of microspheres and can be fabricated through methods such as microfluidics [1]. Fluorescent microspheres function as versatile tracers with applications spanning across biomedicine [2,3], complex plasma systems [4], hydrodynamics [5], and drug delivery [6]. Conventional methods typically encapsulate small-molecule dyes within the microsphere matrix [7]; however, the porous structure of hydrogels frequently results in dye leakage, fluorescence quenching, and uncontrolled release. Consequently, the development of fluorescent microspheres capable of stable encapsulation and controlled release represents a critical challenge that requires urgent resolution.

Intermolecular noncovalent interactions—such as hydrophobic interactions, hydrogen bonding, and π–π stacking—play pivotal roles in the preparation of diverse materials, offering new approaches to fabricate microspheres with stable dye encapsulation. Wei et al. [8] leveraged the hydrogen bond between tannic acid (TA) and polyvinyl alcohol (PVA) to create PVA@TA_1_ hydrogel membranes that can quickly and evenly form a layer for fruit preservation. The coating crosslinked quickly (within 50 s), extending the freshness of the fruit for up to 8 d. Liu et al. [9] employed TA to modify the physicochemical properties of graphene surfaces, thereby improving its adsorption efficiency for RhB. TA-functionalized graphene (TA-G) effectively adsorbs the dye molecule RhB via noncovalent interactions. The synergistic effects of these noncovalent interactions significantly enhance the adsorption capacity, rendering TA-G a promising material for dye wastewater treatment. Tang et al. [10] synthesized TA-functionalized graphene hydrogels (GT) using a one-step hydrothermal method. The introduction of TA markedly improved the adsorption capacity of the graphene hydrogels for methylene blue (MB). The enhancement in adsorption performance was primarily due to noncovalent interactions between TA and methylene blue. Molecular dynamics simulations provide a critical methodological bridge to address these experimental constraints [11]. In molecular dynamics simulations, Newton’s equations of motion are used extensively to simulate the states and trajectories of molecular systems, and samples obtained from molecular systems in various states are used to calculate the configurational integrals of the systems [12]. GROMACS is a popular molecular dynamics simulation program that can be used to determine how macromolecules, polymers, organics, and inorganics interact [13]. The combination of experiments and molecular dynamics simulation (MDS) helps better verify the interactions between molecules. Experiments supply reliable data, while simulations reveal atomic-level mechanisms and dynamic behavior.

Poly(ethylene glycol) diacrylate (PEGDA) has been used extensively in many different industries owing to its inexpensiveness, superior biocompatibility, and modifiable mechanical qualities [14,15]. PEGDA exhibits favorable photopolymerization characteristics. In the presence of an initiator and UV light, PEGDA can be polymerized rapidly. This process offers the benefits of requiring a low initiator dosage and achieving a fast reaction time [16]. Moreover, PEGDA hydrogel microspheres prepared via microfluidic technology are commonly employed as carriers for drugs and cells due to their excellent loading capacity. Lin et al. [17] employed microfluidic fabrication to develop chitosan/PEGDA hydrogel microspheres as a chondrocyte encapsulation platform. Tannic acid (TA), possessing a polyhydroxylated structure, exhibits a high dye adsorption capacity. This capability is attributed to its distinct molecular architecture, which facilitates interactions with diverse dyes via π–π interaction, electrostatic interaction, hydrogen bonding, and other physicochemical interactions. Feng et al. [18] prepared biocarbon-based silver nanoparticle-sodium alginate-tannic acid composite gel beads, which integrated antimicrobial, antioxidant, and dye adsorption capabilities, and the authors found a marked increase in the adsorption performance of dyes on the TA-added gel spheres compared to the gel spheres without the addition of TA, which may be due to the existence of π–π interactions between the dyes and TA. Li et al. [19] modified MoS_2_ using TA, and the resulting TAMoS_2_ nanocomposite film demonstrated quick and excellent selectivity for cationic dyes with various characteristics. TA in TAMoS_2_ nanosheets can efficiently adsorb cationic dyes by trapping the cations via cation–π interactions and direct interactions between hydrated cations and oxidized groups owing to its numerous phenolic hydroxyl groups. Tang et al. [10] prepared a graphene-tannic acid hydrogel from TA, which exhibited the strongest adsorption capacity for the organic dye methylene blue compared to that of reduced graphene oxide hydrogels.

To address the instability of fluorescent dye encapsulation in tracer microspheres, RhB was stably incorporated into PEGDA microspheres by exploiting the noncovalent interactions between TA and RhB. This study proposes the utilization of non-covalent molecular interactions to achieve stable encapsulation of RhB within hydrogel microspheres, thereby preparing tracer microspheres with stably encapsulated dye. Therefore, microfluidic technology was employed in this study to prepare PEGDA microspheres with uniform and controllable particle sizes. PEGDA@RhB-TA microspheres were fabricated by incorporating TA, a distinctive polyphenolic natural compound, into the system, enabling stable encapsulation and responsive release of RhB. The critical function of hydrogen bonding in stabilizing the encapsulation was confirmed by elevating the temperature from 50 °C to 80 °C. This elevated temperature can enhance molecular thermal motion and reduce the hydrogen bonding force between RhB and TA. A change in the quantity of ethanol alters the polarity of the solution, which disrupts the hydrophobic connections between RhB and TA, leading to responsive RhB release. The non-covalent interactions present between TA and RhB stabilized the encapsulation of RhB in PEGDA microspheres. Additionally, MDS was performed using GROMACS to calculate the binding free energies between RhB and TA, etc. The noncovalent interactions between RhB and TA were further confirmed by combining the simulation results with experimental outcomes. It was found that RhB and TA have hydrogen bonding and hydrophobic, π–π, and ionic-π interactions, with the hydrophobic interactions being predominant. Using these noncovalent interactions, RhB and TA make contact and aggregate with one another, stabilizing the encapsulation of RhB in PEGDA microspheres. Following their introduction into a microfluidic chip, the positions of PEGDA@RhB-TA microspheres can be tracked using a high-speed camera and fluorescence microscope to calculate flow velocity, demonstrating their potential for fluid tracing and flow rate measurement applications.

## 2. Materials and Methods

### 2.1. Materials

Poly(ethylene glycol) diacrylate (PEGDA, Mw ~400), Tannic acid (TA, 98%), Rhodamine B (RhB, AR), Methyl Orange (MO, 98%), Isopropanol (purity ≥99.5%), Hexadecane (AR, 98%), 2-Hydroxy-4′-(2-hydroxyethoxy)-2-methylpropiophenone (Photoinitiator-2959, 98%), Span 80 (AR, 99%), Ethyl Alcohol (AR) were obtained from Shanghai Macklin Biochemical Technology Co., Ltd.(Shanghai, China). NaCl (AR, purity ≥99.5%) was obtained from Shanghai Hucheng Laboratory Equipment Co., Ltd.(Shanghai, China).

### 2.2. Preparation of Hydrogel Microspheres

Span 80 solution (10 g) and hexadecane solution (90 g) were weighed. The two were thoroughly combined to create a hexadecane solution with 10 wt% Span 80 as the continuous phase. Subsequently, 0.25 g of Photoinitiator-2959 was dissolved in deionized water (10 g) to create the dispersed phase, before being stirred at 50 °C until the Photoinitiator-2959 was completely dissolved. Following that, PEGDA (1.25 g) was then added to the solution containing the Photoinitiator-2959 and stirred to create a homogenous mixture.

The dispersed phase flow rate was set at 5 μL/min, and the continuous phase flow rate was set at 150 μL/min. The flow-focusing microfluidic chip was utilized, and the droplets were cured into microspheres following UV polymerization. An isopropanol–water solution of a 1:1 volume ratio was created by thoroughly mixing 50 mL of isopropanol solution with 50 mL of deionized water to clean the PEGDA microspheres. The PEGDA microspheres were placed in a centrifuge tube, rinsed with a suitable volume of isopropanol–water solution, and subsequently centrifuged at 1500 rpm. The supernatant was then removed, and the washing process was repeated until the supernatant was clear.

### 2.3. Preparation of PEGDA@RhB-TA Microspheres

RhB (2.395 g) was dissolved in deionized water (100 mL) to make a 0.05 mol/L solution. Another 0.05 mol/L solution was created by dissolving TA (8.5 g) in deionized water (100 mL). A 2 mL aliquot of washed PEGDA microspheres was dispersed in an isopropanol–water solution (V_isopropanol_:V_water_ = 1:1, 10 mL). Following homogenization, 1 mL of the dispersion was transferred to a centrifuge tube using a pipette. The tube was centrifuged at 1500 rpm, followed by removal of the supernatant for subsequent use.

PEGDA microspheres were immersed in 0.5 mL of 0.05 mol/L RhB solution within a centrifuge tube and soaked for 20 min. Following soaking, the supernatant RhB layer was removed using a syringe. Subsequently, the microspheres were soaked in a 0.05 mol/L TA solution (0.5 mL) for 10 min. Following secondary soaking, the microspheres were rinsed repeatedly using 1 mL aliquots of deionized water until a colorless, clear supernatant was achieved, with the supernatant layer being removed after each rinsing.

### 2.4. Preparation of PEGDA@RhB Microspheres

PEGDA microspheres were immersed in 0.5 mL of 0.05 mol/L RhB solution within a centrifuge tube and soaked for 20 min. Following soaking, the supernatant RhB layer was removed using a syringe. Then, the microspheres were rinsed three times using 1 mL aliquots of deionized water, with the supernatant layer being removed after each rinsing.

### 2.5. Preparation of PEGDA@MO-TA Microspheres

Methyl Orange (1.637 g) was dissolved in deionized water (100 mL) to make a 0.05 mol/L solution. PEGDA microspheres were immersed in 0.5 mL of 0.05 mol/L Methyl Orange (MO) solution within a centrifuge tube and soaked for 20 min. Following soaking, the supernatant MO layer was removed using a syringe. Subsequently, the microspheres were soaked in a 0.05 mol/L TA solution (0.5 mL) for 10 min. Following secondary soaking, the microspheres were rinsed repeatedly using 1 mL aliquots of deionized water until a colorless, clear supernatant was achieved, with the supernatant layer being removed after each rinsing.

### 2.6. Scanning Electron Microscopy (SEM) and Fourier Transform Infrared Spectroscopy (FTIR)

FTIR was employed to examine the interactions and functional group composition of PEGDA, RhB, TA, and PEGDA@RhB-TA. The above samples were dried completely, mixed with a suitable amount of KBr, and pressed into pellets for analysis by FTIR spectroscopy. FTIR measurements were performed using a Nicolet iS20 spectrometer (Thermo Fisher Scientific, Waltham, MA, USA). SEM was employed to characterize the interior structures of the PEGDA, PEGDA@TA, and PEGDA@RhB-TA hydrogel microspheres. After freeze-drying, the PEGDA, PEGDA@TA, and PEGDA@RhB-TA hydrogel microspheres were subjected to platinum spraying and then examined using SEM on a ZEISS Gemini SEM 300 instrument (ZEISS, Oberkochen, Germany) operated at an acceleration voltage of 3.00 kV.

### 2.7. RhB Release from PEGDA@RhB-TA Microspheres in Different Salt Solution Concentrations

NaCl (5.88 g) was dissolved in deionized water (100 mL) to create a 1 mol/L solution. This solution was then diluted ten times to create a 100 mmol/L solution. The 100 mmol/L solution was diluted 10 times and 100 times to create 10 mmol/L NaCl solution and 1 mmol/L NaCl solution, respectively. The same quantity of PEGDA@RhB-TA microspheres was added to 5 mL of 1 mol/L, 100 mmol/L, 10 mmol/L, and 1 mmol/L NaCl solution (25 °C), respectively. The release rate of RhB was determined using its standard curve, with absorbance measurements performed at 556 nm over time using UV spectrophotometry.

A standard curve was established by measuring the absorbance of RhB standard solutions at the maximum absorption wavelength of 556 nm. The concentrations of the standards were prepared at 1, 5, 10, 15, 20, 150, and 200 μg/mL, using deionized water as the solvent. Absorbance measurements were carried out with an Agilent Cary 5000 spectrophotometer. The resulting linear calibration curve was expressed as y = 0.00311x + 0.00218 (R^2^ = 0.99923), where y represents absorbance and x denotes the RhB concentration in μg/mL. The standard curve for RhB is shown in the Figure S1.

### 2.8. RhB Release from PEGDA@RhB Microspheres at 10 mM NaCl

PEGDA@RhB microspheres were placed in 5 mL of 10 mmol/L NaCl solution. A UV spectrophotometer was used to measure the absorbance of RhB at 556 nm over time, and a standard curve of RhB was created to determine the release rate of RhB.

### 2.9. RhB Release from PEGDA@RhB-TA Microspheres at Different Temperatures

Four groups of the same quantity of PEGDA@RhB-TA microspheres were placed in 5 mL of 10 mmol/L NaCl solution, after which the four groups of microspheres were heated at 50 °C, 60 °C, 70 °C, and 80 °C, respectively. A UV spectrophotometer was used to measure the absorbance of RhB at 556 nm over time, and a standard curve of RhB was created to determine the release rate of RhB.

### 2.10. RhB Release from PEGDA@RhB-TA Microspheres in Ethanol–Water Solutions with Varying Volume Ratios

Deionized water (100 mL) was combined with 10 mL, 30 mL, and 50 mL of ethanol solutions, respectively, to create 10% *v*/*v*, 30% *v*/*v*, and 50% *v*/*v* ethanol–water mixed solutions. Three identical PEGDA@RhB-TA microsphere sets were each added to 5 mL of a 10% *v*/*v*, 30% *v*/*v*, and 50% *v*/*v* ethanol–water mixed solution. A UV spectrophotometer was used to measure the absorbance of RhB at 556 nm over time, and a standard curve of RhB was created to determine the release rate of RhB.

### 2.11. Hydrodynamics Simulation

The finite element simulation was used to numerically simulate the droplet formation region of the flow-focused microfluidic chip. The two-phase flow-phase field technique, which assumes that the fluid in the channel moves in a laminar flow, was utilized to model the droplet production process of the flow-focused device using the quadratic polynomial (P2) and a primary polynomial (P1) fluid discretization mode [1]. Generally, the P2 and P1 are used to estimate the velocity and pressure fields of a fluid. The P2 + P1 fluid discretization model was selected to balance simulation duration and closeness to real experimental results. The P2 + P1 mode suggests that pressure is solved using a linear basis function, while velocity is solved using a quadratic function. Table S1 presents the precise simulation settings, and Figure S2 in the Supplementary Information displays the boundary conditions.

The experimental viscosity and density, interfacial tension, contact angle, and velocity of the two phases were entered into the finite element software for simulation. The initial conditions of the flow field model were set as an oil-solution-filled flow field with a simulation step size of 1 ns and a time interval of 2000 ns.

### 2.12. Molecular Dynamics Simulation

Chemical software was used to plot PEGDA, TA, and RhB. The B3LYP generalization of the DFT [20] method was used to optimize the small molecule structures in the 6-31g(d) basis set using the Gaussian09 software. Multiwfn [21] used the optimized structures to determine the RESP [22] charges of TA and RhB, and parameters of the force field (GAFF) for small molecules were constructed using the Sobtop software [1.0(dev3)] [23,24,25].

All simulations were performed using GROMACS version 2023.2 [26]. The general properties were described using the Amber99 [27] force field parameterization, and tiny molecules were described using the GAFF force field parameterization. The TIP3P water model was used to fill the system with water molecules after a certain ratio (RhB:TA:PEGDA = 1:1:3.5) was placed into the box with the box boundary at 15 Å from the contents. Energy minimization (1000 kJ/mol/nm) was performed using the steepest descent method, and then a 0.5 ns NPT pre-equilibrium simulation was executed. The following simulation parameters were set: a V-rescale [28] thermostat was utilized, the temperature was set to 298.15 K, and a C-rescale [29] pressure controller was employed to maintain the pressure at 1 bar. Long-range van der Waals interactions were truncated at 10 Å, dispersion correction was performed using EnerPres, and electrostatic interactions were treated using particle-mesh Ewald [30,31]. The trajectories were saved every 5 ps during a 100 ns production simulation with a step size of 2 fs. Trajectory snapshots were acquired using VMD1.9.3 [32], and PyMOL2.5.0 was used to depict the local interaction structure.

Molecular mechanics generalized Born surface area (MM/GBSA) was used to compute the free energy of binding (Δ*G_bind_*) of TA and RhB [33]. The gmx mmpbsa [34] program was utilized to determine the binding energies.

### 2.13. PEGDA@RhB-TA Microsphere Trajectories in Variously Shaped Microfluidic Channels

PEGDA@RhB-TA microspheres were introduced into spiral and U-shaped flow channels, respectively, using a syringe pump. Subsequently, water was introduced into the flow channels, and the microspheres’ trajectories were recorded using a high-speed camera (YA-M1350C, Guangzhou Rongfeng Optoelectronics Technology Co., Ltd., Guangzhou, China) and a fluorescence microscope (XD, Sunny Optical Technology (Group) Co., Ltd., Ningbo, China).

## 3. Results and Discussion

### 3.1. Preparation of PEGDA Microspheres

**Figure 1:**
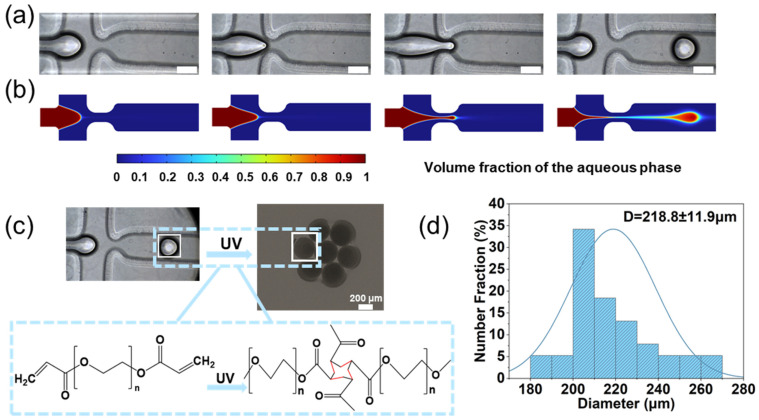
Microfluidic fabrication and characterization of PEGDA microspheres. (**a**) The process of Droplet Generation in Microfluidic Chips. (Scale bar: 200 μm) (**b**) Simulation of droplet production in microfluidic chips. (Contact angle = 90°, Փ = 30, Flow rate: 0.05 mL/min, Aqueous phase: PEGDA) (**c**) The process of droplets into microspheres (The red bonds are the new single bonds formed by opening the original double bonds). (**d**) Diameter distribution of PEGDA microspheres.

PEGDA hydrogel microspheres were created using a flow-focused microfluidic chip (Figure 1a). The droplet formation process can be separated into two stages: growth of the droplet head and shear fracture of the droplet neck, where the continuous phase extrudes the dispersed phase to create droplets. Hydrogel microsphere production is influenced by multiple factors, requiring an integrated approach of numerical simulations and experimental validation to analyze droplet generation dynamics. As depicted in Figure 1b, two-phase flow hydrodynamic modeling simulated PEGDA droplet formation in the microfluidic chip. Comparative analysis with experimental data demonstrated close agreement between simulations and experiments.

Generated droplets were subjected to UV irradiation for solidification (Figure 1c). Controlled exposure induced consolidation into solid microspheres, with photopolymerization-induced crosslinking of PEGDA forming hydrogel networks. The particle size of the hydrogel microspheres was primarily distributed in the range of 210–230 μm, with an average size of 218.84 μm. The PEGDA microspheres produced using this method exhibited uniform and controllable particle sizes, as confirmed by the microsphere count presented in Figure 1d.

### 3.2. Preparation of PEGDA@RhB-TA Microspheres

**Figure 2:**
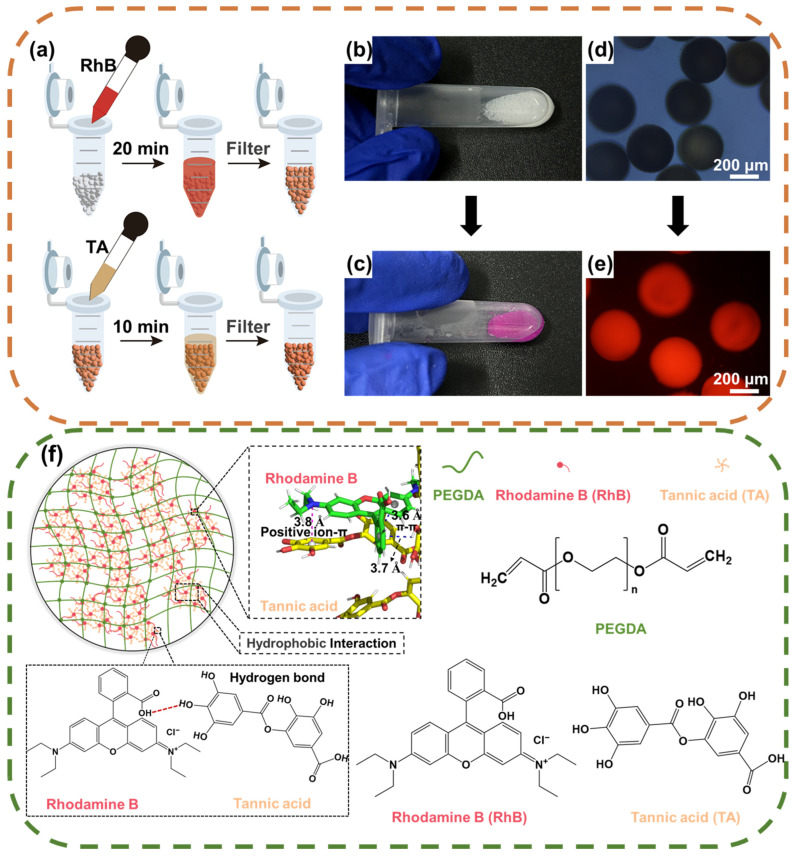
Preparation and characterization of PEGDA@RhB-TA microspheres. (**a**) Schematic of the preparation process. (**b**,**c**) Macroscopic photographs of PEGDA and PEGDA@RhB-TA microspheres, respectively. (**d**,**e**) PEGDA and PEGDA@RhB-TA microspheres observed by fluorescence microscope. (**f**) Schematic of the noncovalent interaction between RhB and TA.

A schematic diagram of the PEGDA@RhB-TA microsphere preparation process is shown in Figure 2a. RhB was incorporated into PEGDA microspheres via immersion-mediated physical adsorption, which resulted in diffusion throughout the polymeric network. Upon submersion of PEGDA@RhB microspheres in TA, hydrophobic interactions between the benzene rings of TA and RhB induced proximity. RhB/TA coloading induced hydrogel pore contraction and network densification in PEGDA microspheres. Upon being exposed to natural light, the PEGDA@RhB-TA microspheres also displayed a red hue, which enhanced the visibility of the microspheres (Figure 2b,c). Furthermore, PEGDA@RhB-TA microspheres exhibited excitation-specific fluorescence uniformity, as depicted in Figure 2d,e.

The noncovalent interaction between RhB and TA is depicted in Figure 2f. Figure 2 2f depicts a key structural unit of TA. The complete molecule comprises multiple such units. π–π interactions occurred between the benzene rings. Concurrently, ion–π interactions formed between the quaternary ammonium cation of RhB and the electron-rich benzene rings of TA. Concurrent hydrogen bonding formed between the phenolic hydroxyl groups of TA and the carboxylic acid residues of RhB. These noncovalent interactions collectively stabilized RhB encapsulation within the PEGDA microspheres. In this process, RhB was securely fixed into PEGDA microspheres via the non-covalent interactions between TA and RhB, which served as “molecular glue”.

### 3.3. SEM and the FTIR Spectra Analysis

**Figure 3:**
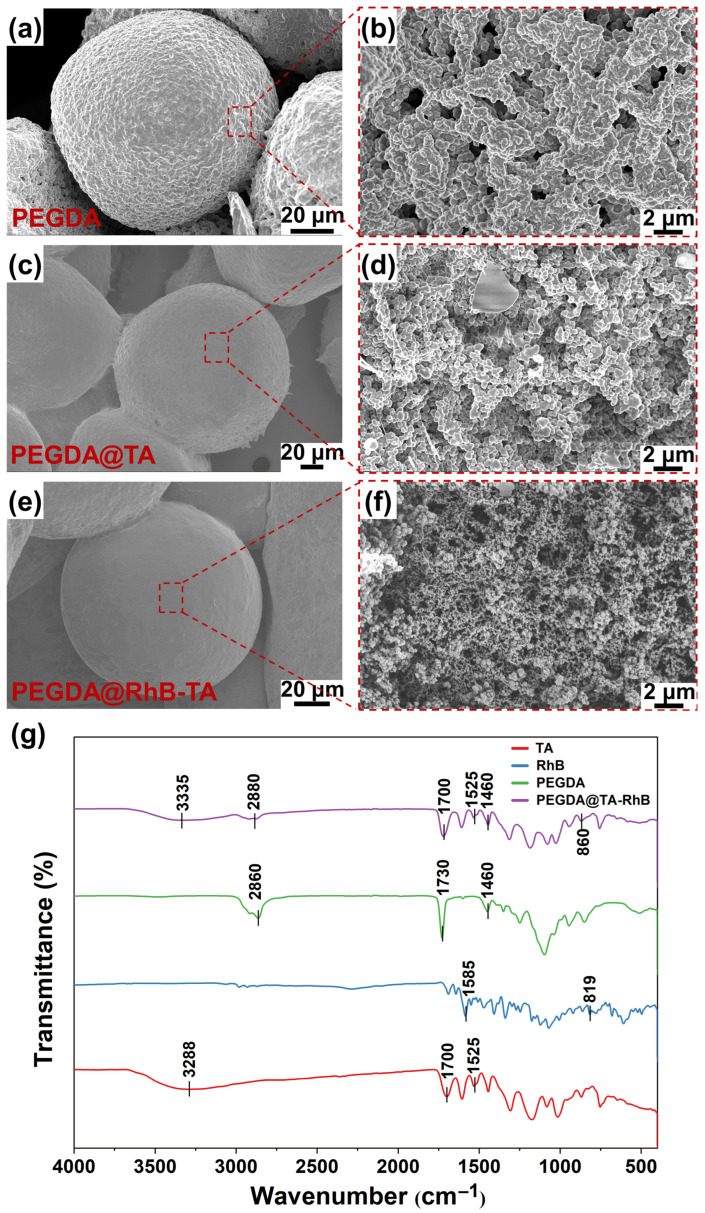
Morphological and chemical characterization of microspheres. (**a**,**c**,**e**) SEM images showing surface morphology, and (**b**,**d**,**f**) pore structure of blank PEGDA, PEGDA@TA, and PEGDA@RhB-TA microspheres, respectively. (**g**) The FTIR spectra of TA, RhB, PEGDA, and PEGDA@RhB-TA.

The pore architectures of PEGDA, PEGDA@TA, and PEGDA@RhB-TA microspheres were characterized via SEM to observe the change in microsphere pore size. The morphologies of blank PEGDA, PEGDA@TA, and PEGDA@RhB-TA microspheres under a scanning electron microscope are displayed in Figure 3a, Figure 3c, and Figure 3e, respectively. The pore structures of blank PEGDA, PEGDA@TA, and PEGDA@RhB-TA microspheres are shown in Figure 3b, Figure 3d, and Figure 3f, respectively. The images reveal significantly larger pore dimensions in blank PEGDA microspheres (1–5 μm width range), whereas PEGDA@TA exhibits intermediate porosity. This indicates that the incorporation of TA partially occupies the PEGDA network, thereby introducing a “matrix effect” that could influence the permeation of RhB through the network to some extent. Minimal pore size and reduced pore density are observed in PEGDA@RhB-TA microspheres. This reduction occurs because RhB first infiltrates the pores of blank PEGDA microspheres upon submersion. Following the addition of TA, RhB and TA are combined via noncovalent interactions, filling the microspheres, and inducing size reduction. Mutual aggregation yields RhB-TA complexes that possess greater bulk dimensions than TA, enabling more effective pore occupation within PEGDA microspheres and decreasing pore diameters.

The FTIR spectra of TA, RhB, blank PEGDA microspheres, and PEGDA@RhB-TA microspheres are illustrated in Figure 3g. The red curve shown in Figure 3g represents the FTIR spectra of TA. The C-C stretching vibration of the benzene ring skeleton, ester group (C=O) vibration, and the hydroxyl (-OH) vibration are responsible for the absorption peaks of TA at 1525, 1700, and 3288 cm^−1^, respectively [35,36]. The broader peak at 3288 cm^−1^ can be attributed to intramolecular hydrogen bonding formed by the nearby hydroxyl groups, as well as intermolecular hydrogen bonding between TA and TA. The development of intermolecular hydrogen bonding in TA is likewise supported by the shift of the -OH absorption peak to lower frequencies. The blue curve shown in Figure 3g displays the FTIR spectra of RhB. The benzene ring skeleton vibration of RhB and the ester group (C=O) vibration cause the absorption maxima at 819 and 1585 cm^−1^, respectively [37]. In Figure 3g, blank PEGDA microspheres’ FTIR spectra are displayed as green curves. The C-H bending vibration of the aliphatic chain in the poly(ethylene glycol) (PEG) chain, vibration of the ester group (C=O), and telescopic vibration of the alkyl groups in the polymer are represented by the absorption peaks of blank PEGDA microspheres at 1460, 1730, and 2860 cm^−1^, respectively [38,39]. The purple curve shown in Figure 3g illustrates the FTIR spectra of PEGDA@RhB-TA. Since the frequency of the absorption peak at 3335 cm^−1^ is slightly higher than that of TA at 3288 cm^−1^, this indicates that hydrogen bonding within the TA molecule is reduced, and TA may be more inclined to form intermolecular hydrogen bonds with RhB, a finding subsequently confirmed in subsequent experiments.

### 3.4. RhB Release from PEGDA@RhB-TA Microspheres in Varying Concentrations of NaCl Solution

**Figure 4:**
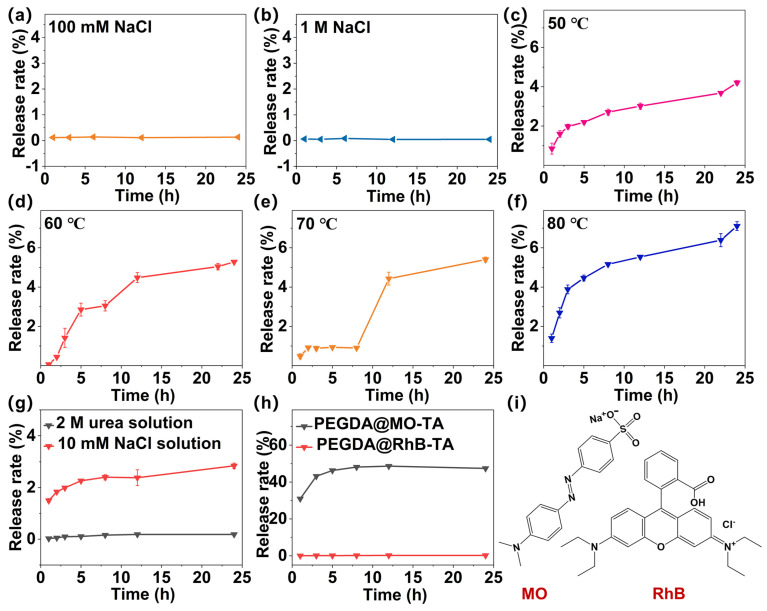
Release rate profiles of RhB from PEGDA@RhB-TA microspheres under different conditions. (**a**,**b**) RhB release in 100 mM and 1 M NaCl. (**c**–**f**) RhB release at 50 °C, 60 °C, 70 °C, 80 °C. (**g**) RhB release in 2 M urea and 10 mM NaCl. (**h**) RhB and MO release from respective microspheres in 10 mM NaCl. (**i**) Molecular structures of MO and RhB.

Equal volumes of PEGDA@RhB-TA microspheres were submerged in NaCl solutions at concentrations of 1 mM, 10 mM, 100 mM, and 1 M, to counteract electrostatic interference (25 °C). This procedure was performed to allow the positive charge in the RhB structure and negative charge in the TA structure to be neutralized by Na^+^ and Cl^−^ in NaCl, respectively. The release rate profiles of RhB in PEGDA@RhB-TA microspheres in 1 M and 100 mM NaCl solutions are displayed in Figure 4a and Figure 4b (within 24 h), respectively. Figure S3 presents the release rate profiles of RhB from PEGDA@RhB-TA microspheres over 48 h in NaCl solutions at concentrations of 1 mM, 10 mM, 100 mM, and 1 M. These profiles were ascertained using the RhB standard curve. In aqueous solution, TA exhibits anionic character owing to partial ionization of its phenolic hydroxyl groups, enabling electrostatic interaction with cationic structures [40]. Contrastingly, RhB is a cationic dye whose aqueous ionization yields colored cations. Consequently, electrostatic attraction between TA and RhB was hypothesized to stabilize RhB encapsulated in PEGDA microspheres. The absence of RhB leakage during 48 h immersion in varied NaCl concentrations indicates that electrostatic interactions do not play a major role in maintaining RhB encapsulation within PEGDA microspheres. This is likely because TA does not deprotonate many hydroxyl groups in neutral conditions, which prevents it from forming significant electrostatic contacts with positively charged RhB.

The release profiles of RhB from PEGDA@RhB and PEGDA@RhB-TA microspheres under identical conditions (10 mM NaCl) are presented in Figure S4. The results demonstrate that when RhB was solely loaded into PEGDA microspheres, the release rate reached 73% within 1 h and approached equilibrium at approximately 88% after 5 h. These findings indicate that PEGDA microspheres alone cannot effectively encapsulate RhB. Comparative analysis of the release profiles between PEGDA@RhB and PEGDA@RhB-TA under the same experimental conditions revealed a significantly higher release rate from PEGDA@RhB than from PEGDA@RhB-TA. This observation clearly demonstrates that the incorporation of TA substantially improves the encapsulation efficiency of RhB. The moisture content of PEGDA microspheres, PEGDA@TA microspheres, and PEGDA@RhB-TA microspheres was measured simultaneously. The results demonstrated moisture contents of 8.54% for PEGDA microspheres, 6.37% for PEGDA@TA microspheres, and 4.54% for PEGDA@RhB-TA microspheres. The PEGDA@RhB-TA microspheres exhibited significantly lower water content compared to both PEGDA and PEGDA@TA microspheres. These findings indicate that the enhanced crosslinking density in PEGDA@RhB-TA microspheres reduces the network’s water absorption capacity. Furthermore, the high crosslinking density and reduced pore size of PEGDA@RhB-TA microspheres may explain the observed difficulty in RhB release.

### 3.5. Release of RhB from PEGDA@RhB-TA Microspheres at Different Temperatures

Minimal influence of electrostatic interactions on RhB release from PEGDA@RhB-TA microspheres was verified; consequently, NaCl solution (10 mM) immersion was employed to observe RhB release. Cumulative RhB release profiles from PEGDA@RhB-TA microspheres at 50 °C, 60 °C, 70 °C, and 80 °C are presented in Figure 4c–f. Release rates were calculated using a RhB standard calibration curve. Figure 4c–f demonstrate that RhB release rates increase over time at constant temperatures. Elevated temperatures accelerated release rates throughout the 24 h observation period, with cumulative releases reaching 7.1% at 80 °C versus 4.2% at 50 °C after 24 h. Stabilization of RhB encapsulation is facilitated by hydrogen bonding interactions between multiple hydroxyl groups of TA [41] and the carboxyl group of RhB. Thermal treatment enhances molecular mobility while weakening hydrogen bonding, thereby increasing the probability that the hydrogen bond will break. Consequently, when the temperature increases, RhB can be released, and the rate of release of RhB increases with the temperature.

The microspheres were immersed in a 2 M urea solution to further verify the role of hydrogen bonding in RhB encapsulation stabilization. The RhB release profile from PEGDA@RhB-TA microspheres in 2 M urea is presented in Figure 4g, demonstrating that microspheres immersed in 2 M urea solution exhibited approximately 3% RhB release at 24 h, whereas negligible RhB release was observed for microspheres immersed in urea-free solution. Urea addition was performed to disrupt hydrogen bonding between RhB and TA, as urea has been characterized as a potent hydrogen bond disrupter [42]. The results clearly showed that hydrogen bonding plays a significant role in stabilizing the encapsulated RhB. The release rate profiles of RhB in PEGDA@RhB-TA microspheres and MO in PEGDA@MO-TA microspheres in 10 mM NaCl are presented in Figure 4h. Figure 4h shows that MO can be released normally and that it reaches release equilibrium after 8 h. No hydrogen bond will form between MO and TA because, unlike RhB, the MO molecule lacks a carboxyl group that would enable it to do so, as shown in Figure 4i. In summary, Figure 4g,h provide indirect evidence that the hydrogen bond formed between the TA group and RhB is crucial for the stable encapsulation of RhB within PEGDA microspheres.

### 3.6. RhB Release from PEGDA@RhB-TA Microspheres in Various Volume Ratios of Ethanol and Water

**Figure 5:**
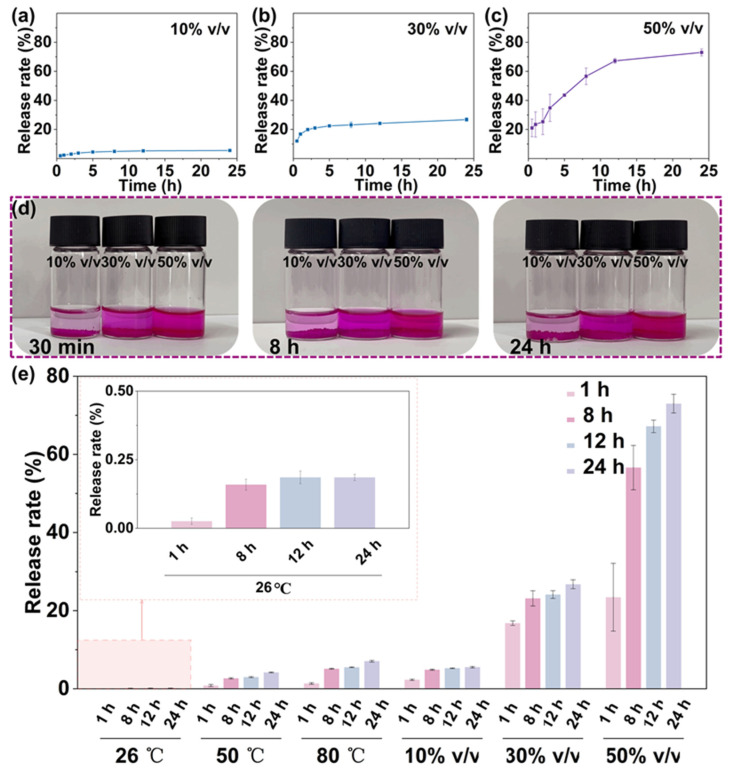
Effect of ethanol concentration on RhB release. (**a**–**c**) Release profiles at 10%, 30%, and 50% ethanol (*v*/*v*). (**d**) Images of PEGDA@RhB-TA microspheres during release. (**e**) RhB release rates from PEGDA@RhB-TA microspheres in different conditions.

PEGDA@RhB-TA microspheres were immersed in ethanol–water solutions of varying volume ratios, and the RhB content in the supernatant was quantified. Release rate profiles for RhB in 10% *v*/*v*, 30% *v*/*v*, and 50% *v*/*v* ethanol–water solutions are shown in Figure 5a–c. Increased RhB release rates were observed over time at both constant ethanol concentrations and increasing ethanol concentrations. When PEGDA@RhB-TA microspheres were submerged in a 50% *v*/*v* ethanol–water solution for 24 h, the release rate of RhB reached 73%, which was 13.15 and 2.72 times higher than the release rate of RhB submerged in 10% *v*/*v* and 30% *v*/*v* ethanol–water solutions, respectively. Figure 5d shows a considerable rise in RhB release as the ethanol concentration increases. Considerable RhB release was rapidly achieved; at 30 min, 44% and 28.8% of total RhB release were measured for microspheres submerged in 30% *v*/*v* and 50% *v*/*v* ethanol–water solutions, respectively. The structural composition of RhB features hydrophilic carboxyl groups alongside hydrophobic benzene rings and ethyl groups, whereas TA possesses hydrophobic benzene rings, ester bonds, and hydrophilic hydroxyl groups. Hydrophobic interactions between RhB and TA are speculated to occur, defined as the propensity of nonpolar molecular fragments to group in aqueous media [43,44]. Within the hydrophilic networks of PEGDA microspheres, hydrophobic segments of TA and RhB are brought into proximity owing to water molecule repulsion, establishing hydrophobic interactions that stabilize RhB encapsulation. Solution polarity critically influences this stabilization, where ethanol addition modifies the polarity [45]. The rapid disruption of TA-RhB hydrophobic interactions occurs upon polarity alteration, enabling RhB release responsiveness.

Figure 5e shows the release rates of RhB from PEGDA@RhB-TA microspheres under different conditions for better comparison. As shown in the Figure 5e, when the release time is 24 h, almost no RhB is released at room temperature (26 °C), followed by the least release when heated at 50 °C, followed by the release from microspheres immersed in 10% *v*/*v* ethanol-aqueous solution, with intermediate release when heated at 80 °C, and a significant increase in the release of RhB observed when immersed in 30% *v*/*v* and 50% *v*/*v* ethanol-aqueous solutions. In contrast, PEGDA@RhB-TA microspheres immersed in 50% *v*/*v* ethanol–water solution released 17.4 times and 10.3 times more RhB than those heated to 50 °C and 80 °C, respectively. This result demonstrates that hydrophobic contacts and hydrogen bonds are crucial for maintaining the stability of encapsulated RhB.

### 3.7. Molecular Dynamics Simulation

**Figure 6:**
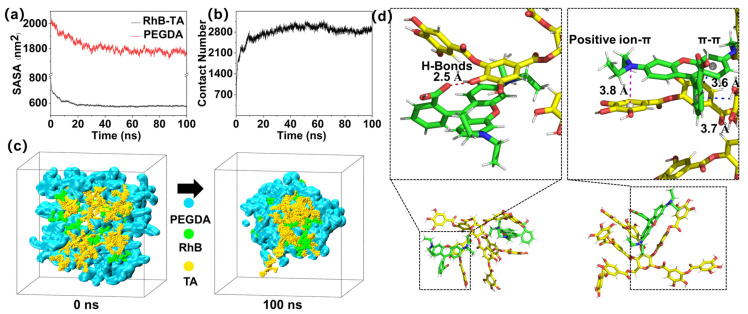
Molecular dynamics simulations of RhB and TA (**a**) SASA analysis of PEGDA and RhB-TA. (**b**) Contact number analysis of RhB and TA nonpolar C atoms (<0.5 nm). (**c**) Polymer profiles at 0 ns and 100 ns. (**d**) Interactions between the systems RhB and TA. Key: hydrogen bonds (red dashed lines), π–π interactions (blue), ion–π interactions (magenta), TA (Yellow Structure), RhB (Green Structure), PEGDA (Blue Structure).

To further validate the mechanism for the stable encapsulation of RhB, MDS were performed to qualitatively investigate the noncovalent interactions between RhB and TA. The low-crosslinking model in this simulation serves as a preliminary approximation of the actual crosslinking network environment, while the binding behavior of RhB and TA under real network constraints is determined by analyzing experimental data. Figure S5 shows the molecular structures of PEGDA, RhB, and TA. Figure 6a illustrates the variation in the solvent accessible surface area (SASA) of PEGDA and RhB-TA with simulation time. SASA quantifies the molecular surface area exposed to water. A reduction in SASA corresponds to enhanced intermolecular associations, and the attainment of a plateau value indicates interaction stability. Consequently, monitoring SASA facilitates the characterization of hydrophobic interactions [46]. A progressive reduction in SASA values is observed for PEGDA and RhB-TA as simulation time increases. This observation suggests decreased contact areas of PEGDA, TA, and RhB with the aqueous solvent. SASA values of RhB and TA remain essentially unchanged beyond 20 ns, indicating the formation of stable interactions between RhB and TA as well as the presence of a hydrophobic interaction. Stabilization of the SASA value of PEGDA is likewise observed after 60 ns, confirming structural stability of the system and formation of stable nanoclusters. Contact numbers of hydrophobic carbon atoms (predominantly benzyl carbons) on RhB and TA molecules were additionally evaluated (Figure 6b). Stabilization of contact numbers was observed beyond 20 ns, indicating strong intermolecular binding. This result supports the hypotheses regarding potential π–π interactions, hydrophobic interactions, and additional interaction modalities between RhB and TA. Radial distribution function (RDF) analysis, a method for characterizing the relative atomic/molecular distance distributions to study interaction types and strengths, was conducted between RhB and hydrophobic carbon atoms of TA [47]. As shown in Figure S6a, RDF peaks are observed at 0.49 nm with significant distribution between 0.3–6 nm, indicative of concurrent π–π and ion–π interactions (ion– interactions occur at 0.4–0.5 nm; π–π interactions within 0.4 nm). Hydrogen bonding between RhB and TA was subsequently investigated (Figure S6b). A gradual increase in the hydrogen bond count was observed before 20 ns, followed by stabilization beyond this timeframe, with bond counts stabilizing within the 6–12 range. This stabilization confirms hydrogen bonding between RhB and TA.

Snapshots of the general structure of the kinetic simulations at 0, 0.5, 2.5, 20, 60, and 100 ns are presented in Figure S7. At 0 ns, molecules are randomly dispersed within the box. Between 0.5 and 20 ns, molecules establish contact, leading to incomplete interactions. Figure S7 shows that PEGDA molecules form localized structures, while RhB and TA are observed in proximity, interacting through the gaps in the PEGDA network. At 20 ns, the majority of the RhB and TA are in contact with one another and are progressively enclosed by PEGDA, causing the overall size to diminish. From 20 to 60 ns, the overall clusters gradually alter in shape, while numerous small clusters are still visible. The system reaches its final stable shape between 60–100 ns, when the presence of some free PEGDA fragments stabilizes the main cluster structure. The overall clusters are displayed in the section (Figure 6c). RhB and TA are more widely distributed in the system at 0 ns, and PEGDA is present between the two substances. The stable structure is formed at 100 ns, and the distribution of PEGDA between RhB and TA decreases. RhB and TA are in contact with one another, which prevents PEGDA from reaching the outer part of the clusters, forming the encapsulated morphology.

Molecular interactions among system components were characterized by schematically representing interfacial bonding structures. Hydrogen bonding formation between RhB and TA is visualized in Figure 6d. Additionally, π–π stacking interactions between aromatic rings and cation–π interactions between the positively charged nitrogen of RhB and the phenyl rings of TA were observed. Therefore, stabilization of RhB and TA within the crosslinked PEGDA network was achieved via various interactions.

The binding free energy of RhB and TA in the last 4 ns is presented in Table 1, and the following equations were used to calculate the binding energy.

(1) $$∆G_{bind}=∆H-T∆S\approx ∆G_{solv}+∆G_{GAS}-T∆S$$

(2) $$∆G_{GAS}=∆E_{vdW}+∆E_{ele}$$

(3) $$∆G_{solv}=∆E_{psolv}+∆E_{nsolv}$$

where Δ*G_GAS_* denotes the difference in kinetic energy in the vacuum before and after the binding of the receptor and ligand, which is subdivided into *E_vdw_* and *E_elec_*. *E_vdw_* represents the van der Waals energy change before and after the binding, and *E_elec_* represents the electrostatic energy change. Δ*G_solv_* denotes the free energy of solvation, which is divided into the polar solvation energy Δ*E_psolv_* and nonpolar solvation energy Δ*E_nsolv_*, and −*T*Δ*S* indicates the entropy change, which reflects the energy loss during system binding owing to flexibility or system stability [34,48].

The calculated binding free energy is −400.50 kcal/mol (derived from interactions among multiple components). The system contains 30 RhB molecules and 30 TA molecules. The interaction energy for each RhB-TA molecular pair is −13.35 kcal/mol. This result supports the experimental finding that the binding of RhB to TA is a spontaneous process. Here, the van der Waals value (Δ*E_vdW_*) is −654.13 kcal/mol, which likely arises from the π–π interaction between the benzene rings of RhB and TA, as well as the dispersion force associated with their hydrophobic aggregation (this dispersion component of the van der Waals interaction helps stabilize the hydrophobic assembly of RhB and TA). This result indicates potential hydrophobic interactions or π-π stacking between RhB and TA. The electrostatic energy (Δ*E_ele_*) of −756.82 kcal/mol indicates that RhB and TA have a significant charge complementarity, while hydrogen bonding is a directional charge attraction [49], suggesting that RhB and TA may form a hydrogen bond. In addition, the electrostatic values also show ion–π interactions. Additionally, the electrostatic values also show ion–π interactions, showing that RhB and TA may be both hydrogen-bonded and ion–π interacting. Since the polar solvation energy (∆*E_psolv_*) is positive and is opposite in sign to the electrostatic energy (∆*E_ele_*), it is likely counterproductive to the stable encapsulation of RhB. This is likely owing to the water molecules shielding the charged groups, which means that electrostatic interactions are greatly reduced in aqueous solutions [50]. Hydrophobic interactions are advantageous for the stable encapsulation of RhB, as evidenced by the nonpolar solvation energy (∆*E_nsolv_*) being negative and positively proportional to the SASA. The hydrophobic interaction plays a principal role in stabilizing the encapsulation of RhB within microspheres, based on the combined free energy and experimental results. Lastly, the contribution of various RhB and TA to the binding energy was noted, as illustrated in Figure S8. The horizontal axis denotes specific RhB and TA molecules. The vertical axis quantifies the strength of intermolecular noncovalent interactions for each molecule, where more negative values correspond to stronger interactions. Computational analysis revealed that most RhB molecules and some of the TA molecules tend to form non-covalent interactions with other molecules.

Therefore, MDS revealed that RhB and TA aggregate within PEGDA via multiple noncovalent interactions. The combined experimental and MDS results demonstrate that hydrophobic interactions are the dominant factor in stabilizing RhB encapsulation within microspheres.

### 3.8. PEGDA@RhB-TA Microsphere Trajectories in Variously Shaped Microfluidic Channels

**Figure 7:**
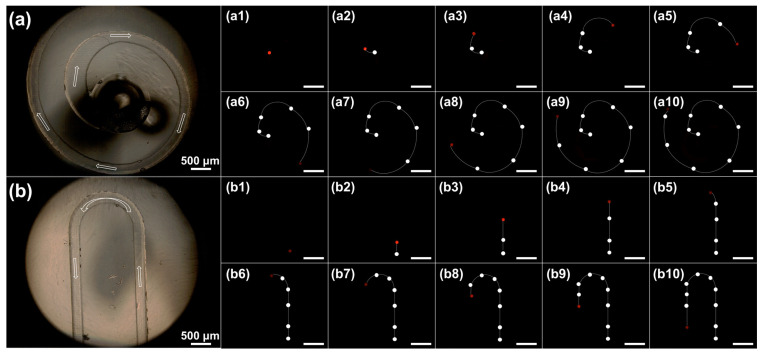
Trajectory analysis of PEGDA@RhB-TA microspheres in spiral and U-shaped flow channels. (**a**,**b**) Schematics of the channels. (**a1**–**a10**) Trajectories of PEGDA@RhB-TA microspheres in the spiral flow channel. (**b1**–**b10**) Trajectories of PEGDA@RhB-TA microspheres in the U-shaped flow channel. The scale bar is 1 mm.

The motion trajectories of the microspheres were analyzed using a fluorescent microscope after PEGDA@RhB-TA microspheres were passed into two distinct flow channels. The morphology of the spiral and U-shaped flow channels under the microscope is depicted in Figure 7a and Figure 7b, respectively. The trajectories of PEGDA@RhB-TA microspheres within the spiral flow channel are depicted in Figure 7(a1–a10), whereas those within the U-shaped flow channel are displayed in Figure 7(b1–b10). The red ball depicts the immediate position of the PEGDA@RhB-TA microspheres in the figure, while the trajectory of the PEGDA@RhB-TA microspheres is shown in white. This characteristic enables one to observe that the microspheres can move smoothly along the flow channel, and their trajectory route is a spiral helix and a U-shaped curve. Considering the high-speed camera has a frame rate of 30 fps and the microsphere is 105 frames away from Figure 7(b1–b4), Formula (4) can be used to determine that the time gap between two frames is 0.03 s. Hence, the microsphere moves 3.50 s from Figure 7(b1–b4), covering 2.37 mm in that time. The average velocity of the microsphere across this distance was calculated to be 0.68 mm/s. It is demonstrated that in addition to the acquisition of PEGDA@RhB-TA microsphere trajectories by the optical system, fluid average velocity can be determined from the trajectories. Optical detection systems enable precise capture of fluorescent signals, facilitating the conversion of time-resolved displacement measurements into quantitative velocity data. This demonstrates that optical systems can not only capture the motion trajectories of PEGDA@RhB-TA microspheres within the microfluidic chip but also enable the calculation of the average fluid velocity based on these trajectories, thereby providing flow velocity information.

(4) FPS=1/Time(s)

## 4. Conclusions

A PEGDA@RhB-TA microsphere with stabilized encapsulation and responsive RhB release was successfully fabricated in this study. It was demonstrated that the encapsulation of RhB within PEGDA microspheres was sustained by the non-covalent interactions between TA and RhB, with a predominant role being played by hydrophobic interactions. Minimal RhB release was observed at 80 °C, and no release occurred in high-concentration NaCl solution. However, the addition of ethanol altered the polarity of the solution, disrupting the hydrophobic interactions between RhB and TA, thereby enabling the significant release of RhB. PEGDA@RhB-TA microspheres immersed in a 50% *v*/*v* ethanol–water solution for 24 h demonstrated 73% RhB release. Therefore, responsive RhB release occurs under ethanol exposure. MDS simulations of noncovalent interactions between RhB and TA were performed using GROMACS to further validate these interactions at the molecular level. The stable encapsulation of RhB within PEGDA microspheres (in the absence of disruption to the noncovalent interactions between RhB and TA, there is no release of RhB) is primarily attributed to the non-covalent interactions between RhB and TA, as identified through MDS results and experimental findings. Among these interactions, hydrophobic forces play a dominant role. In summary, this study successfully developed PEGDA-based microspheres that enable the efficient co-loading for the first time and controlled release of the model dye RhB and TA. When introduced into a microfluidic chip, the positions of the PEGDA@RhB-TA microspheres can be tracked using a high-speed camera and fluorescence microscopy, allowing flow velocity to be calculated. These microspheres address the dual challenges of tracer stability and controlled release, rendering them suitable for fluid tracing and flow-velocity measurement. Furthermore, when environmental factors disrupt the non-covalent interactions between RhB and TA, the release of RhB is triggered, thereby providing insight into the chemical microenvironment within the fluid.

## 5. Future Perspectives and Limitations

This study was designed to synthesize PEGDA@RhB-TA microspheres using RhB as a model dye, verifying the stable encapsulation and responsive release capabilities of the microspheres for RhB. Conventional tracers typically provide only physical flow velocity information [4], whereas the PEGDA@RhB-TA microspheres innovatively integrate the functions of flow tracing and chemical sensing. On one hand, flow velocity can be determined by tracking the trajectories of the microspheres using a high-speed imaging system. On the other hand, when the microspheres encounter specific chemical environments (such as ethanol), PEGDA@RhB-TA microspheres release RhB to generate a fluorescent signal, thereby simultaneously marking the location of chemical stimulation. This dual functionality makes PEGDA@RhB-TA microspheres a powerful tool for studying microfluidic chips and aquatic environments. Despite these advantages, the PEGDA@RhB-TA microspheres still exhibit limitations such as irreversible responsiveness and potential susceptibility to specific interference. At the same time, the selection of RhB as a model compound is based on its ease of monitoring and stability, serving as both a drug simulant and a tracer [51]. This study not only demonstrates the potential of PEGDA@RhB-TA microspheres for tracing applications but also shows that their encapsulation strategy can be extended to the loading and controlled release of structurally similar drugs. However, attention must be paid to the differences in molecular properties between RhB and actual drugs, and their release kinetics and therapeutic efficacy require further validation.

## Figures and Tables

**Table 1:** Binding free energies of RhB and TA at the last 4 ns (kcal/mol).

	Δ*E_vdW_*	Δ*E_ele_*	∆*E_psolv_*	∆*E_nsolv_*	∆*G_bind_*
Ave	−654.13	−756.82	1088.27	−77.82	−400.50

## Data Availability

The original contributions presented in this study are included in the article. Further inquiries can be directed to the corresponding author.

## Supplementary Materials

The following supporting information can be downloaded at https://www.mdpi.com/article/10.3390/polym18040493/s1: Table S1: Parameters of the solution condition used in this study; Figure S1: Standard Curve for RhB; Figure S2: Boundary conditions in the droplet generation region; Figure S3: (a–c) Release rate profiles of RhB from PEGDA@RhB-TA microspheres in 1 mM, 10 mM, 100 mM, and 1 M NaCl solutions; Figure S4: (a) Release curve of RhB from PEGDA@RhB microspheres in 10 mM NaCl; (b) Comparison of release rates of PEGDA@RhB microspheres and PEGDA@RhB-TA microspheres RhB from microspheres in 10 mM NaCl; Figure S5: Small molecule structure diagram. (a) PEGDA, (b) RhB, (c) TA; Figure S6: (a) RDF of RhB and TA nonpolar C atoms. (b) Hydrogen bonding analysis between RhB and TA; Figure S7: Simulated structural snapshots at 0, 0.5, 2.5, 10, 60, and 100 ns. Green represents RhB, yellow represents TA, and the polymer is represented as a solvated surface model in the figure; Figure S8: Energy contribution per molecule between RhB and TA.
