# Supplementary material for: Stable Encapsulation and Responsive Release of Dyes via Noncovalent Molecular Lock Strategy: A Case Study of Rhodamine B Based Fluorescent Hydrogel Microspheres
Source: Polymers (Basel). 2026 Feb 16;18(4):493. doi: 10.3390/polym18040493 (PMC12944488; doi:10.3390/polym18040493)
Supplement: Supplementary file 1 [file polymers-18-00493-s001.zip › polymers-4102310-supplementary.pdf]

Supplementary Material for

Stable Encapsulation and Responsive Release of Dyes via Noncovalent Molecular Lock Strategy: A Case Study of Rhodamine B Based Fluorescent Hydrogel Microspheres

Shuo Meng[a], Chuanyu Dang[b], Xiaoyong Qiu[a], Jianhua Chen[c], Ruiheng Yao[d], Yuquan Wang[d], Luxing Wei[d], Jun Huang[d], Xiaolai Zhang[a, *]

[a] Key Laboratory of Colloid and Interface Chemistry of the Ministry of Education, School of Chemistry and Chemical Engineering, Shandong University, Jinan, Shandong 250100, China

[b] Shandong Kunda Biotech Co., Ltd., Linyi, Shandong 276400, China

[c] Beijing Fleming Technology Co., Ltd., Beijing 102600, China

[d] Center for Advanced Jet Engineering Technologies (CaJET), Key Laboratory of High Efficiency and Clean Mechanical Manufacture of Ministry of Education, School of Mechanical Engineering, Shandong University, Jinan, Shandong 25006, China

**Corresponding author: zhangxlai@sdu.edu.cn*

Table S1. Parameters of the solution condition used in this study

Parameters	Value
The flow rate of aqueous phase (mL/min)	0.005
The flow rate of oil phase (mL/min)	0.15
Wall contact angle (°)	90
The viscosity of aqueous solution (Pa·s)	0.00198
The density of the aqueous solution (kg/m³)	1013
The viscosity of oil solution (Pa·s)	0.00045
The density of the oil solution (kg/m³)	773
Interfacial tension (N/m)	0.0176

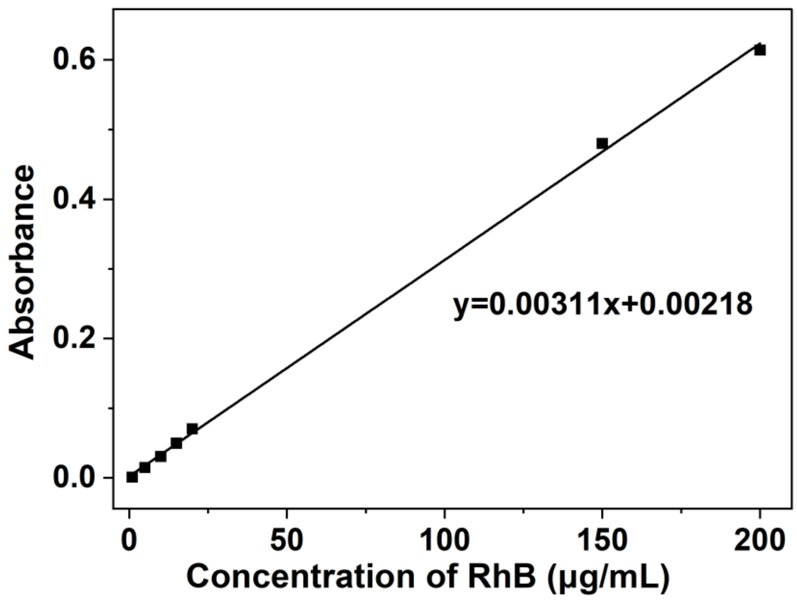

Figure S1. Standard Curve for RhB (Absorbance measured at 556 nm).

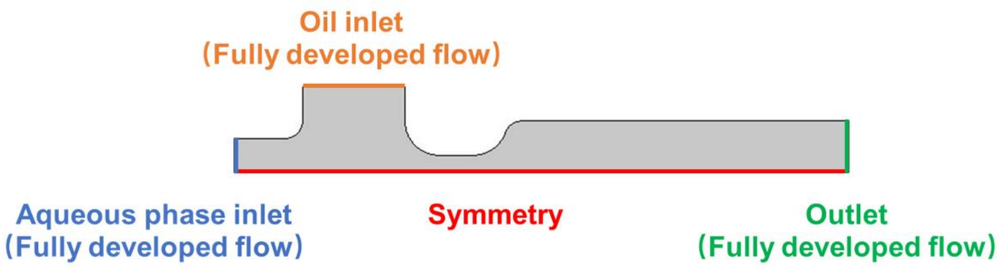

Figure S2. Boundary conditions in the droplet generation region.

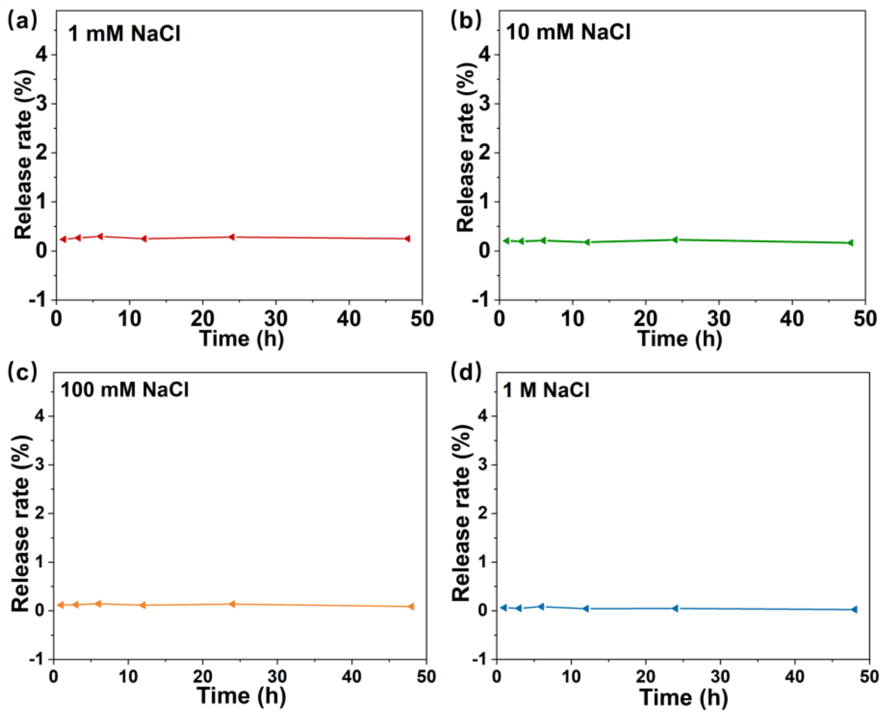

Figure S3. (a-d) Release rate profiles of RhB from PEGDA@RhB-TA microspheres in 1 mM, 10 mM, 100 mM, and 1 M NaCl solutions.

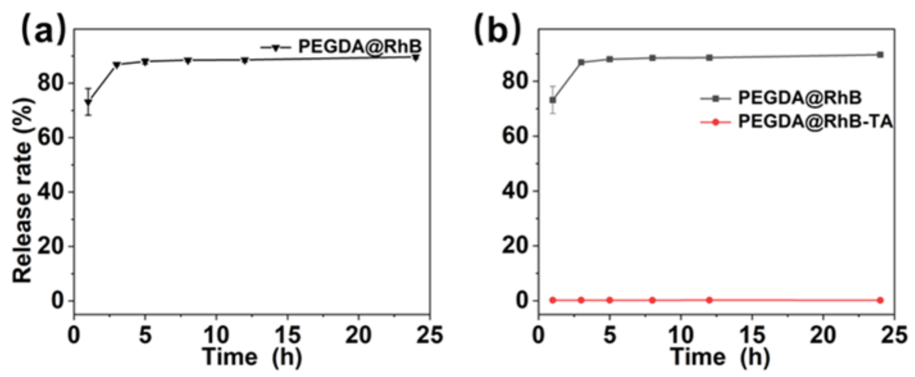

Figure S4. Release curve of RhB from PEGDA@RhB microspheres in 10 mM NaCl; Comparison of

release rates of PEGDA@RhB microspheres and PEGDA@RhB-TA microspheres of RhB from microspheres in 10 mM NaCl.

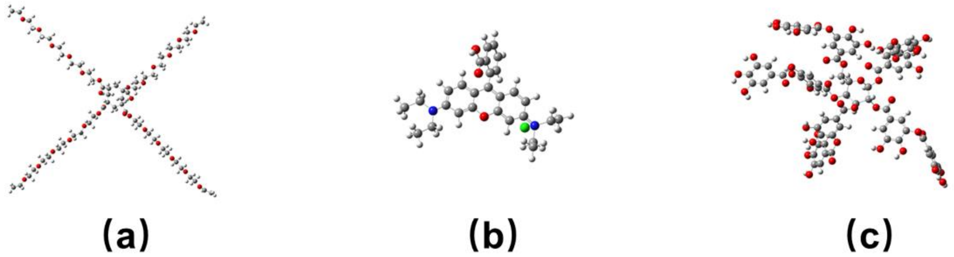

Figure S5. Small molecule structure diagram. (a) PEGDA, (b) RhB, (c) TA.

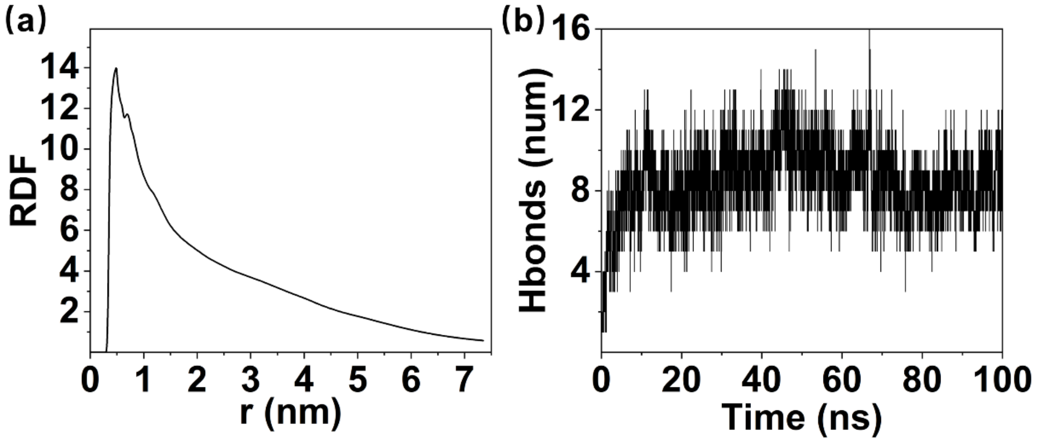

Figure S6. (a) RDF of RhB and TA nonpolar C atoms. (b) Hydrogen bonding analysis between RhB and TA.

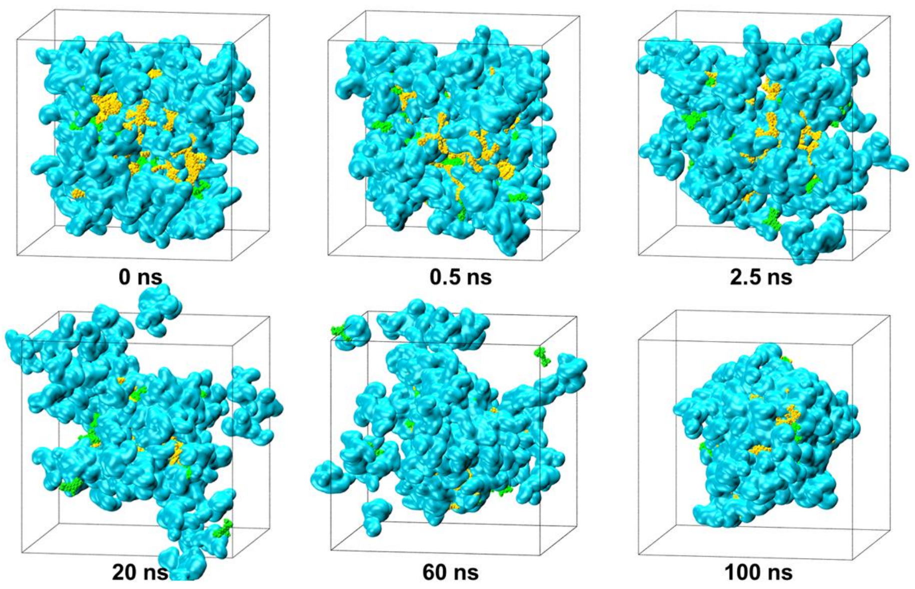

Figure S7. Simulated structural snapshots at 0, 0.5, 2.5, 10, 60, and 100 ns. Green represents RhB,

yellow represents TA, and the polymer is represented as a solvated surface model in the figure.

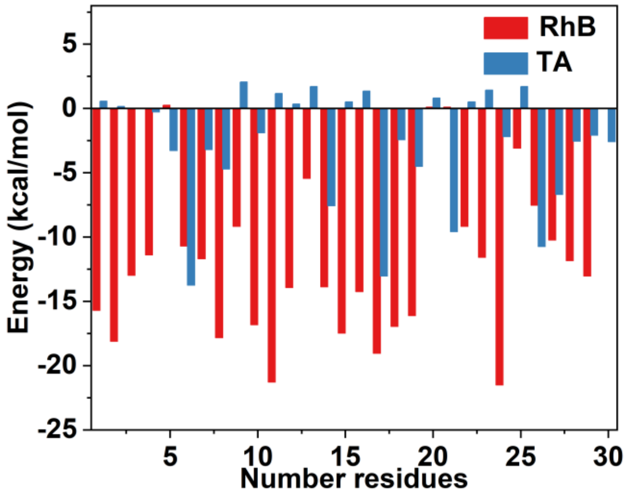

Figure S8. Energy contribution per molecule between RhB and TA.
